# Three New Scorpion Chloride Channel Toxins as Potential Anti-Cancer Drugs: Computational Prediction of The Interactions With Hmmp-2 by Docking and Steered Molecular Dynamics Simulations

**DOI:** 10.22037/ijpr.2019.1100659

**Published:** 2019

**Authors:** Masoumeh Baradaran, Amir Jalali, Maryam Naderi Soorki, Mahmoud Jokar, Hamid Galehdari

**Affiliations:** a *Toxicology Research Center, Ahvaz Jundishapur University of Medical Sciences, Ahvaz, Iran.*; b *Department of Toxicology, School of Pharmacy and Toxicology Research Center, Ahvaz Jundishapur University of Medical Sciences, Ahvaz, Iran. *; c *Department of Pharmacology and Toxicology, School of Pharmacy, Guilan University of Medical Sciences, Rasht, Iran. *; d *Genetics Department, Sciences Faculty, Shahid Chamran University of Ahvaz, Ahvaz, Iran.*; e *Cotton Research Institute of Iran, Agricultural Research, Education and Extension Organization (AREEO), Gorgan, Iran.*

**Keywords:** Chloride channel toxin, MDFF simulation, docking, Iranian *Mesobuthus eupeus*, Steered Molecular Dynamics Simulations

## Abstract

Scorpion venom is a rich source of toxins which have great potential to develop new therapeutic agents. Scorpion chloride channel toxins (ClTxs), such as Chlorotoxin selectively inhibit human Matrix Methaloproteinase-2 (hMMP-2). The inhibitors of hMMP-2 have potential use in cancer therapy. Three new ClTxs, meuCl14, meuCl15 and meuCl16, derived from the venom transcriptome of Iranian scorpion, *M. eupeus* (*Buthidea* family), show high sequence identity (71.4%) with Chlorotoxin. Here, 3-D homology model of new ClTxs were constructed. The models were optimized by Molecular Dynamics simulation based on MDFF (molecular dynamics flexible fitting) method. New ClTxs indicate the presence of CSαβ folding of other scorpion toxins. A docking followed by steered molecular dynamics (SMD) simulations to investigate the interactions of meuCl14, meuCl15, and meuCl16 with hMMP-2 was applied. The current study creates a correlation between the unbinding force and the inhibition activities of meuCl14, meuCl15 and meuCl16 to shed some insights as to which toxin may be used as a drug deliverer. To this aim, SMD simulations using Constant Force Pulling method were carried out. The SMD provided useful details related to the changes of electrostatic, van de Waals (vdW), and hydrogen-bonding (H-bonding) interactions between ligands and receptor during the pathway of unbinding. According to SMD results, the interaction of hMMP-2 with meuCl14 is more stable. In addition, Arginine residue was found to contribute signiﬁcantly in interaction of ClTxs with hMMP-2. All in all, the present study is a dynamical approach whose results are capable of being implemented in structure-based drug design.

## Introduction

Scorpions venom contains a complex mixture of substances, mainly composed of proteins and peptides, along with lipids, amines and other small molecules, by which they are able to kill or paralyze their prey and protect themselves against predators (1). Some of the venom peptides (toxins) modify the function of ion channels (sodium, potassium and chloride) while some of the others have antimicrobial, hemolytic activity; also, it can be assumed that the larger proteins are mostly enzymes (2). Due to interaction of toxins with a wide variety of pharmacological targets, they are considered as an interesting research field. Some of the scorpion toxins are supposed to lead the way towards the next generation of cancer-fighting drugs (3). Chlorotoxin, a short-chain, disulfide-containing chloride channel toxin identified from the venom of the scorpion *Leiurus quinquestriatus *(*Buthidea* family) (4), has been introduced as a potential agent in cancer therapy (5-7). Chlorotoxin induces paralysis in insects or other invertebrates stung by the scorpion, but no evidence of toxicity has been found in vertebrates. This indicates that the binding of Chlorotoxin on its cell surface receptor has no cell-toxic effects or unwanted physiological consequences, as observed for many other animals’ toxins (8).

Chlorotoxin, unlike the other related scorpion toxins, does not bind directly to the chloride channel; instead, it specifically binds to hMMP-2, on the surface of the cells, as a primary receptor site (9).

h-MMPs, a family of zinc-dependent and calcium-dependent endopeptidases, are responsible for remodeling the extracellular matrix (ECM) (10). These enzymes, by degradation of the ECM, allow cancer cells to migrate out of the primary tumor to form metastasis (11). Therefore, h-MMPs have crucial role in tumor invasion, angiogenesis, and metastasis. Increased expression and activity levels of h-MMPs have been reported in many human tumor cells. 

Currently, 22 family members of h-MMPs have been detected in humans (10). Among all identified h-MMPs, hMMP-2 (gelatinase A) is thought to play a key role in degradation of the main collagen components of the ECM (12). A significant increase in hMMP-2 expression has also been documented to correlate with tumor aggression and cancer invasion in many experimental and clinical studies (13-18). 

Some studies have discovered that Chlorotoxin, through targeting the hMMP-2, is effective against the spread of tumors in some cancers including glioma, melanoma, small cell lung carcinoma, neuroblastoma, and medulloblastoma by disabling their metastatic activity (6, 19). Accordingly, natural type or synthetically engineered types of Chlorotoxin have been proposed for use in cancer drug delivery systems (6, 7); Chlorotoxin-conjugated nanoparticles have been utilized for targeted imaging (20) or surgically removing of the cancerous tissues (21). Desirable features of Chlorotoxin and Chlorotoxin-like peptides (such as ClTx-a, b, c, d, BmKCL1, Lqh-8:6, Be I5A, BeI1, AmmP2 and GaTx1) have led to the screening of other scorpion venoms with the aim to identify Chlorotoxin-like peptides (6). Thanks to their effectiveness against different tumors, it is believed that the scorpion-derived Chlorotoxin-like peptides can be utilized in synthesis of new specific drugs (22).

A review of the literature shows that only a few chloride channel blockers or Chlorotoxin-like peptides have been identiﬁed from scorpions or other fauna during the last two decades (9). In this paper, 3-D structures of three new Chlorotoxin-like ClTxs, identified from the transcriptome of Iranian *M. eupeus* venom, were predicted by adopting homology modeling followed by employing MDFF simulation to optimization the structures. *in silico* interactions of the ClTxs with hMMP-2 were elucidated through molecular docking process. Binding affinity of ligand-receptor complexes has been evaluated by the Steered Molecular Dynamics (SMD) method (23). The SMD is one of the various recent successful approaches for the calculation of binding free energies of biomolecules (24). 

Predicting the binding free energy of ligands attached to macromolecules can be of great practical value in identifying novel molecules that can bind to target receptors and act as therapeutic drugs (23). In SMD experiments, several pulls are simulated in one (forward) or two (forward and reverse) directions (25). Furthermore, with keeping some group of atoms fixed (receptor), study of the behavior of a protein under various conditions is possible. It has been pointed out that the SMD method has the potential to specify the binding energy of protein–ligand complexes and distinguish strong binders from weak ones (26-29). Thus, here, the SMD method, as a reliable tool, was applied to identify the more active ligand (ClTx).

## Experimental

*cDNA library construction and amino acid sequence determination of ClTxs*

A full-length cDNA library was prepared from the total RNA extracted from the venom glands of *M. eupeus, *collected from the southwestern province of Iran, using the In-Fusion™ SMARTer™ cDNA Library Construction Kit (CLONTECH Lab., Palo Alto, CA) as described elsewhere. Following the cDNA cloning into the pSMART2IFD Linearized Vector, the recombinant vectors were transformed into the bacterial host to prepare the cDNA library (30). The complete cDNA sequence of ClTxs was analyzed by ORF-finder program (http://www.ncbi.nlm.nih.gov/projects/gorf/). The sequence of ORFs was confirmed with protein BLAST program on NCBI (http://www.ncbi.nlm.nih.gov/) and UniProt (http://www.uniprot.org/) servers. The confirmed sequences of the three ClTxs (meuCl14, meuCl15 and meuCl16) submitted to GenBank under certain accession numbers (KU316183, KU316184 and KU316185). Signal peptides were predicted by signal P4.1 available on http://www.cbs.dtu.dk/services/SignalP/. 

*Modeling*

*Homology Model of ClTxs*

The primary structure of new ClTxs shares high sequence identity (71.4%) with Chlorotoxin (according to BlastP). To model their 3D structure, a template based on the most similar toxin to ClTxs that has a crystal or solution nuclear magnetic resonance structure. A search for similar sequences using the BLAST program against the Protein Data Bank Proteins (PDB) database revealed 76% identity with the sequence of InsectotoxinI5A (PDB ID: 1SIS), whose nuclear magnetic resonance structure has been submitted. Homology modeling was performed with the program MODELLER for calculation of the 3-D models of ClTxs taken from the Iranian *M. eupeus* transcriptome (31). 

**Figure 1:**
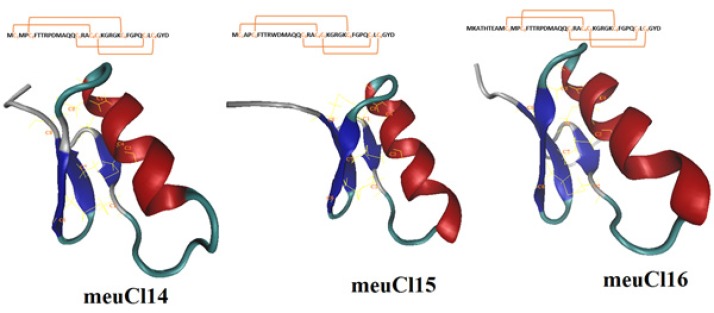
3-D models of meuCl14, meuCl15 and meuCl16 predicted by MODELLER and after simulation with MDFF method. Disulfide bonds (yellow) in meuCl14, meuCl15 and meuCl16 connect β–sheet to α-helix facilitating the folding of peptides into a CSαβ structure

**Figure 2:**
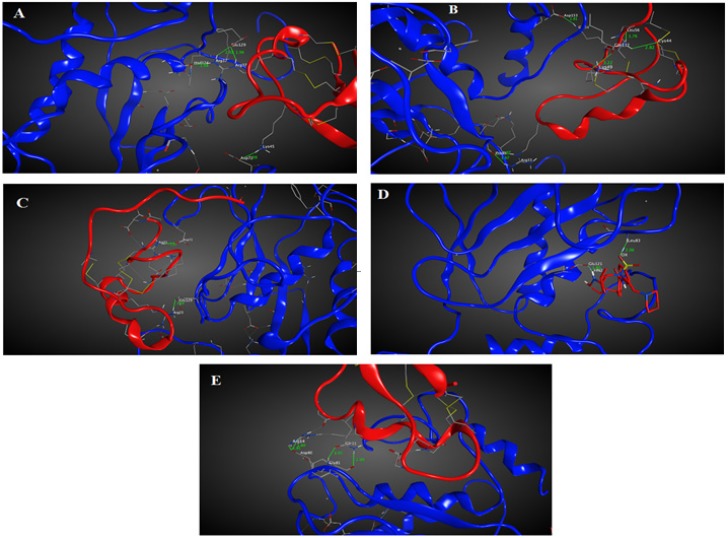
Ribbon models of interaction of MMP-2 catalytic domain with (A) meuCl14, (B) meuCl15, (C) meuCl16 and (D) SC-74020

(E) Chlorotoxin, after SMD simulation and energy minimization. MMP-2 is indicated in blue, ClTxs and SC-74020 are shown with red. The amino acid residues of H-bond contacts between MMP-2 catalytic domain and ClTxs and SC-74020 are shown. Disulfide bonds in ClTxs are shown with yellow lines.

**Figure 3:**
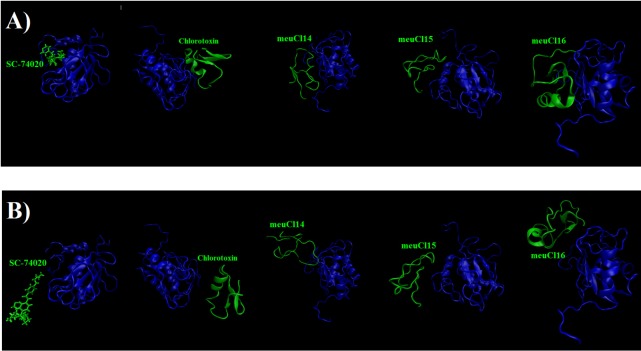
(A) The initial structure of the SMD simulation of ClTx-MMP-2 and SC-74020- MMP-2 complexes (simulation in 0ns). (B) Final structures of the steered molecular dynamics simulations of ClTx-MMP-2 and SC-74020- MMP-2 complexes (simulation in 10ns). hMMP-2 catalytic domain is indicated in blue, ClTx and SC-74020 are shown with green

**Figure 4:**
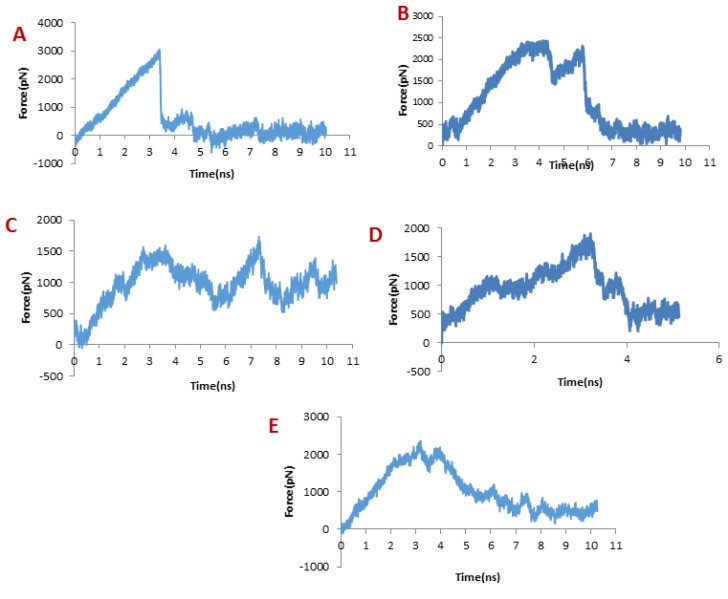
Pulling force for dissociation of ligands from hMMP-2 catalytic domain. Pulling force for unbinding of (A) SC-74020, (B) Chlorotoxin, (C) meuCl14, (D) meuCl15, (E) meuCl16

**Figure 5:**
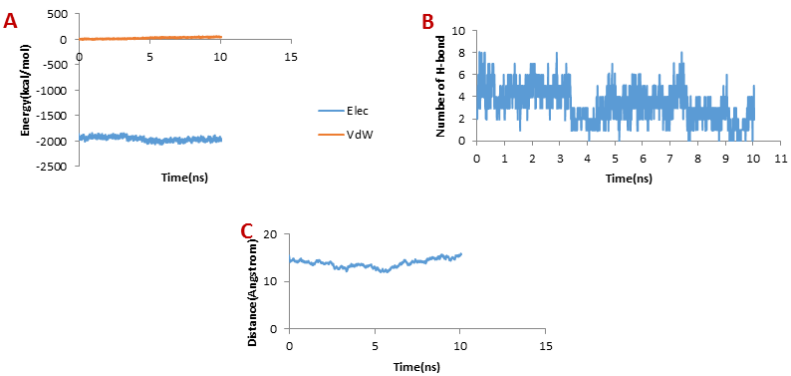
(A) Changes of vdW and electrostatic interaction energy between SC-74020 and hMMP-2 catalytic domain during dissociation. (B) Changes of the number of H-bonds interaction between SC-74020 and hMMP-2 catalytic domain during dissociation.

(C) Distance between SC-74020 and hMMP-2 catalytic domain during dissociation of the SC-74020.

**Figure 6:**
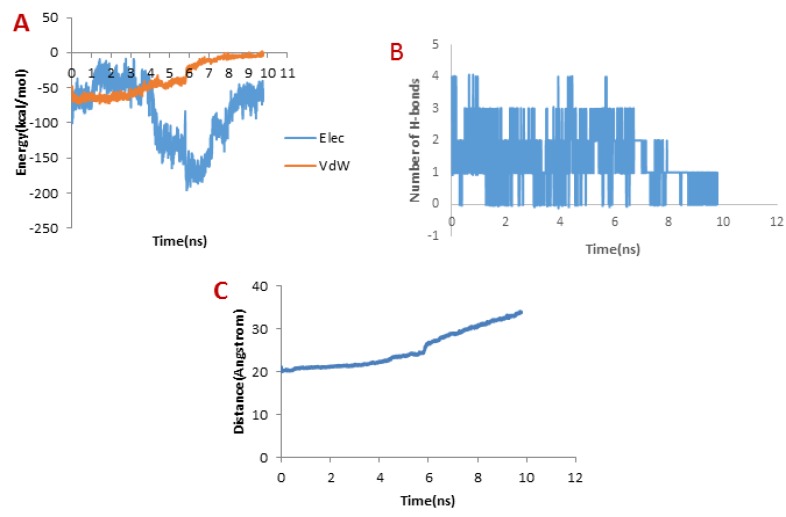
(A) Changes of vdW and electrostatic interaction energy between Chlorotoxin and hMMP-2 catalytic domain during dissociation. (B) Changes of the number of H-bonds between Chlorotoxin and hMMP-2 catalytic domain during dissociation. (C) Distance between Chlorotoxin and hMMP-2 catalytic domain during dissociation of the Chlorotoxin

**Figure 7:**
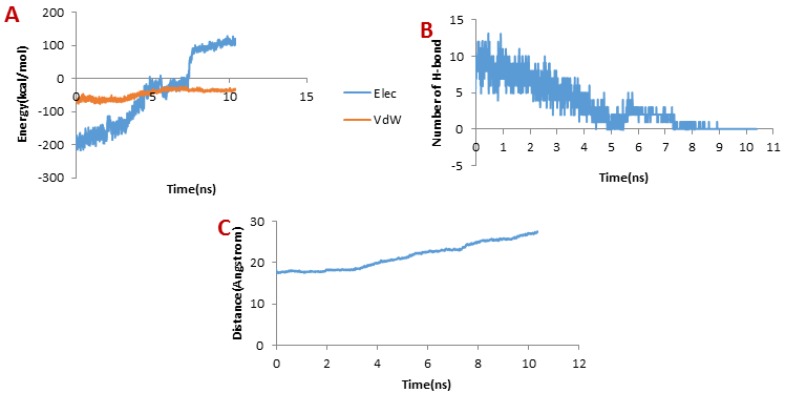
A) Changes of vdW and electrostatic interaction energy between meuCl14 and hMMP-2 catalytic domain during dissociation

(B) Changes of the number of H-bonds between meuCl14 and hMMP-2 catalytic domain during dissociation. (C) Distance between meuCl14 and hMMP-2 catalytic domain during dissociation of the meuCl14.

**Figure 8:**
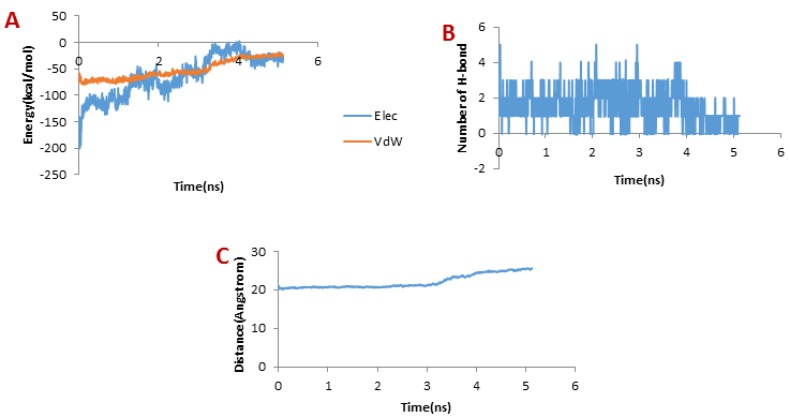
(A) Changes of vdW and electrostatic interaction energy between meuCl15 and hMMP-2 catalytic domain during dissociation

**Figure 9:**
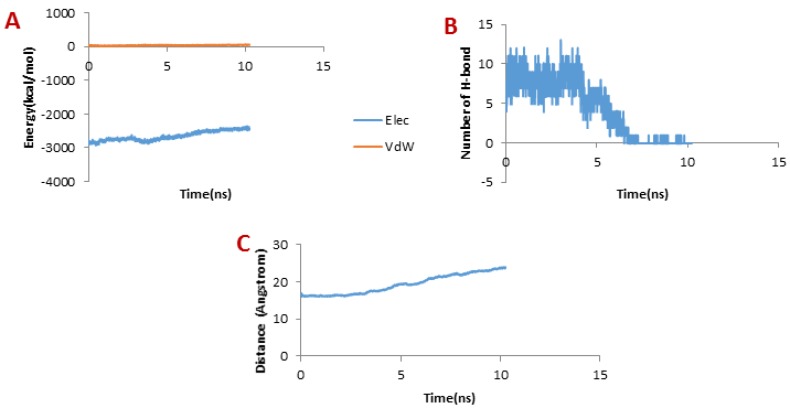
**(**A) Changes of vdW and electrostatic interaction energy between meuCl16 and hMMP-2 catalytic domain during dissociation

(B) Changes of the number of H- bonds interaction between meuCl16 and hMMP-2 catalytic domain during dissociation. (C) Distance between meuCl16 and hMMP-2 catalytic domain during dissociation of the meuCl16.

*Homology models refinement*

MDFF simulations were performed to optimize the obtained homology models. MDFF method was put into practice so as to merge the structures stemming from the homology modeling with the energy maps of template crystallographic structures. The refined protein models were centered and immersed in a cubic box water (triple point charge: TIP3P) by applying the periodic boundary conditions in a way that the complex was 10 Å away from each wall. 

The possible negative charge in the system was neutralized by sodium chloride (NaCl). The Particle Mesh Ewald (PME) method was implemented to accurately estimate the electrostatic interactions. A 10 (Å) cut-off was applied to Lennard-Jones interactions. While running the simulations, in order to hold the temperature and pressure constant for all components, the V-rescale coupling algorithm was employed (32). 

The energy minimization was carried out utilizing the 10,000 steepest-descent algorithm; then, water and ions equilibrated for 50 ps through position-constrained molecular dynamics (MD) simulation, and were simulated under isothermal-isobaric (NVT) ensemble for 200 ps. Finally, the ultimate complex simulations were run under the canonical isothermal-isochoric (NPT) ensemble with the help of NAMD 2.9 (33, 34) with a time step of 2 fs for 20 ns (10 million steps). CHARMM 27 was utilized to parameterize (35). VMD software was employed to visualize the trajectories and their analyses, to wit: RMSD and RMSF, radius of gyration and solvent accessible surface area (SASA) 

(36).

*Simulation ClTx/hMMP-2 interaction in silico*

*Computational Docking*

Three new modeled ClTx structures also with NMR structure of Chlorotoxin (PDB code: 1CHL), as control; and 3D structure of hMMP-2 catalytic domain (PDB ID: 1hov), as receptor (PDB ID: 1hov) were chosen for ligand-receptor docking. 

Hydroxamic acid inhibitor (SC-74020) (common ligand name I52, PDB ID: 1HOV), a standard inhibitor ligand of hMMP-2, was employed as the second control molecule in all steps.

The interaction of predicted model of ClTxs (ligand) with hMMP-2 (receptor) was docked with flexible side chains of hMMP-2 using induced fit protocol by MOE software. In order to detect the active site and bonding properties, the interaction between SC-74020 and the residues of hMMP-2 was set as reference model. Next, docking analyses were performed for ClTxs/hMMP-2 complexes. Triangle Matcher was put into practice for the Placement method, London dG for Rescoring 1. The final scoring was carried out through the GBVI/WSAdG scoring function. 

After the docking procedure, the most frequent conformation in the first cluster of docking results was selected to evaluate the complex stability by the SMD molecular dynamics. 

*SMD simulation*

In order to evaluate the binding interaction between hMMP-2 and ClTxs (meuCl14, meuCl15 and meuCl16), the ClTx/hMMP-2 complexes were simulated to dissociate the ligands from their receptors with the SMD simulation by Constant Force Pulling. In this method, the backbone atoms of the hMMP-2 were kept fixed, while the backbone of every ClTx experienced a constant force in the direction defined by x, y, and z components of the normalized direction between the fixed and the SMD atom. The simulation time for each SMD simulation was 10 ns.

All the simulations were performed with molecular dynamics simulation program NAMD (34), and the CHARAMM27 force field (37). Before the SMD simulation, each complex was embedded in a square periodic box, in which the shortest distance between the protein surface and the box’s walls was larger than 10 Å. The box was filled with water molecules represented by the simple point charge model (38). At the same time, favorable counter ions such as Na^+^ or Cl^−^ were added into the box so as to balance the net charge of the system. The system was energy-minimized without constraints with the steepest descent method, and then 100 ps position-restrained molecular dynamics at 300 K and 1.0 bar was performed to make sure the equilibration of the solvent molecules and ligands with the protein was maintained. In this run, the atom positions of the receptor were restrained and the backbone of receptor was fixed to restrict their movement in the simulation. Next, 100 ps MD simulation without position-restraint was performed to ensure the equilibration of the system. Finally, the ligand was pulled with an external force in the NPT ensemble at 1.0 bar and 300K with 2 fs time steps. Force direction will adjust automatically the center of the mass of the receptor-peptides. Direction axis for meuCl14, meuCl15, meuCl16, and control molecule were (0.2646, -0.1449, 0.9533), (-0.3447, -0.4014, 0.8485), (0.3412, -0.6589, 0.6703) and (-0.6000, 0.2578, -0.7572). The force constant of the spring was 8.5 kcal/mol/Å^2^, and the rate at which the spring was pulled was 10 Å/ns. When the minimum distance between the atoms of the receptor and the ligand reached a cut-off value of 0.5 nm, the corresponding time was recorded as the ending time of the dissociation simulation.

## Results and Discussion

The full length cDNA of meuCl14, meuCl15, and meuCl16, identified from the venom gland of Iranian *M. eupeus*, are related to the ClTxs. ClTxs are small peptides that block conductive chloride channels (22, 39). Mature peptide of meuCl14 and meuCl15 composed of 36 amino acids, while mature peptide of meucl16 contains 44 amino acids. Figure 1S (supplementary materials) shows the amino acid alignment of meuCl14, meuCl15, and meuCl16 with the Chlorotoxin and Chlorotoxin-like peptides family identified in other scorpions. Here identified ClTxs demonstrate 71.4% sequence identity with Chlorotoxin extracted from *Leiurus quinquestriatus *venom, known as a small 36-amino-acids peptide (4).

Homology modeling predicted the 3-D structure of meuCl14, meuCl15, and meuCl16 based on the primary alignment to the known structure of InsectotoxinI5A (PDB code: 1SIS) (amino acid alignments are shown in Figure 1S). The MDFF method has successfully been applied for optimizing the models obtained from the MODELLER. Figure 1 illustrates the predicted models for the ClTxs at the end of the simulation. meuCl14, meuCl15 and meuCl16 contain eight cysteines forming four disulfide bridges, and present two structural domains: one alpha-helix and a triple stranded beta-sheet, indicating that they adopt a typical CSαβ folding. CSαβ structure is composed of a single α-helix connecting to a double or triple stranded β-sheet through four disulfide bonds, identified as a conserved motif in many scorpion toxins (40). Disulfide bonds of meuCl14, meuCl15, and meuCl16 were stable during the simulation (Figure 2S). Other analysis of trajectories including RMSD, RMSF, radius of gyration and SASA, calculated for meuCl14, meuCl15, and meuCl16, are shown in Figure 3S-6S, respectively.

Chlorotoxin inhibits the hMMP-2 indicating the importance of this toxin in cancers associated with hMMP-2 activity (19). Hence, the here obtained homology model structures of ClTxs also with Chlorotoxin and SC-74020 (as control) were docked to hMMP-2. Figure 1A-E shows the docking results after energy minimization. As shown in the model, Arg_14_ and Gln_11_ of Chlorotoxin interact with hMMP-2 through hydrogen bonds (Figure 2A and Table 1). MeuCl14 forms four H-bonds through two residues including Lys_45_ and Arg_37_ with several residues of hMMP-2 catalytic domain (Figure 2B and Table 1). MeuCl15 interacts with hMMP-2 catalytic domain residues via six H-bonds. Glu_54_, Leu_56_, Lys_49_, Cys_44_, and Arg_33_ of meuCl15 seem to be the key contacts between the meuCl15 and hMMP-2 (Figure 2C and Table 1). MeuCl16 bonds to hMMP-2 by two Key residues, Arg_31_ and Arg_25_ (Figure 2D, and Table 1). A comparison between the amino acids involved in the interaction in different ClTxs indicates that Arginine residue has a pivotal role in the interaction with hMMP-2 (Figure 2, and Table 1).

To evaluate the affinity of the ClTxs for hMMP-2, the SMD simulation was done successfully on all five complexes with Constant Force Pulling method. Before performing the SMD simulations, to pull ligands (ClTxs and SC-74020) out of hMMP-2 catalytic domain (receptor), energy minimization and equilibration of the whole system (at the designated temperature, pressure and ion contents) were first carried out. Similarly, all complexes were run up to 10 ns to understand how ligands (ClTxs and SC-74020) behave in the equilibration state when hMMP-2 (receptor) remained fixed; in this process, the ligands were pulled during simulation. Figure 3 shows the position of ligands through the hMMP-2 in 0 ns and 10 ns. 

Obviously, increased force appears when the ligands begin to move out of the binding site, regardless of the initial structure and the direction of pulling force, which implies that the ligand encounters energy barriers. When one bond dissociates, the force somehow decreases and then increases for dissociate another bond. So force fluctuations are shown in the force plot, in which the peaks appear when a significant number of bonds dissociate (41).

In dissociation of SC-74020 from the hMMP-2 catalytic domain, the main peak occurred at about 3 ns (see Figure 4A). Eventually, the pulling force became zero at around 4 ns, indicating that the SC-74020 has completely dissociated from the receptor.

The pulling force plots for dissociation of ClTxs from the hMMP-2 are shown in Figure 4B to 4E. Very similar to the SC-74020, the forces increase in the beginning of ClTxs unbinding, but unlike the SC-74020, more than one peak emerged in the pulling force plots of ClTxs. The main peaks for Chlorotoxin appeared at about 4 and 6 ns, for the meuCl15 between 1ns and 4ns, and for the meuCl16 between 3ns and 4ns. Finally, the fluctuations of pulling force for the meuCl15 and meuCl16 decrease after around 4 ns and for the Chlorotoxin after 6ns close to zero, demonstrating their complete dissociation from the hMMP-2 (Figure 4B, 4D, 4E). The main peaks for meuCl14 emerged after 3 ns to 8 ns. But, some peaks still appeared until the ending time of SMD simulation. It means that meuCl14 still resists some forces and needs a little more time for a complete dissociation from the hMMP-2 (Figure 4B). These results visibly indicate that the SC-74020 as compared to ClTxs dissociates easier from the hMMP-2. Moreover, dissociation of meuCl14 from hMMP-2 is more difficult than the dissociation of Chlorotoxin, meuCl15 and meuCl16. 

In order to identify the contributory factors leading to the easy dissociation of SC-74020 and, on the other hand, the difficult dissociation of ClTxs through the hMMP-2 catalytic domain, the key components of interaction energies between each ligand and hMMP-2 catalytic domain during the SMD simulation were examined, including electrostatic, vdW, and H-bonding interactions.

Figure 5A illustrates the changes of the vdW interaction and electrostatic energy during the dissociation of SC-74020 through the hMMP-2 catalytic domain. No changes in the vdW and the electrostatic interaction energies of SC-74020/hMMP-2 complex were observed during the SMD simulations. In the H-bond diagram (Figure 5B), however, apparent fluctuations could be found during the dissociation of SC-74020 through the hMMP-2 catalytic domain. The most of H-bonds dissociated at about 3 ns, which was consistent with the moment when the main peak appeared on the pulling force plot in Figure 2A, implying that the pulling force helped unbind the H-bonds. Ultimately, the H-bonds reached zero, referring to the complete dissociation of SC-74020 through the hMMP-2 catalytic domain. Decreasing the distance of SC-74020 from the hMMP-2 catalytic domain with time can clearly be observed in distance plot until 5 ns, and after that, the distance begins to increase (Figure 5C). So, unbinding process in SC-74020/hMMP-2 complex was performed in two phases: (a) ejecting form catalytic region (0-5 ns), and (b) complete release form the receptor (5-10ns). Figure 3B also plainly shows that the SC-74020 dissociates completely from the hMMP-2 at the end of the SMD simulation (10 ns). 

Considering Figure 6B, the trend for Chlorotoxin was such that the number of H-bonds significantly decreased at about 4 ns; and almost all of H-bonds were dissociated in 6 ns. Therefore, the emergence of the peaks in the force plot at 4 and 6 ns can be attributed to an increase in the force resulting in the breakage of the H-bonds. Figure 6A shows vdW and electrostatic interaction energy during the dissociation of Chlorotoxin through the hMMP-2 catalytic domain. The vdW energy went up continuously from the beginning of the simulation up to 6 ns. It means that the vdW energy had an influential role in this interaction. After this time point, the vdW energy drew nearer zero. The electrostatic interaction energy increased over the first 4 ns interval of the simulation, then declined and again grew at around 6 ns. In other words, at 4 and 6 ns, the most electrostatic interaction energy formed between the Chlorotoxin and hMMP-2; regarding the pull plot, the forces increased in these time points so as to counteract the electrostatic interaction.

Figure 6C, the distance graph, demonstrates that before 4 ns the distance between the Chlorotoxin and the hMMP-2 catalytic domain was fixed. From 4 ns to 6 ns, the distance increased slightly. After 6 ns onward, the distance increased significantly that means the dissociation of Chlorotoxin from hMMP-2. As just said, the interaction of Chlorotoxin with the hMMP-2 catalytic domain involves all H-bonds, vdW, and electrostatic interactions, but the contribution of H-bonds and vdW interactions are more substantial. 

When it comes to meuCl14, as depicted in Figure 7B, the H-bond plot presents obvious fluctuation during the dissociation of meuCl14 from the hMMP-2 catalytic domain. Clearly, after 3 ns, the number of H-bonds is mainly reduced. Hence, the main peak emerges on the pulling force plot at the same moment (see Figure 4C). The second main drop in H-bonds diagram took place at the time point near 7.3 ns, which is in agreement with the time of appearing the second peak on the pulling force plot. After 8 ns, the number of H-bonds returned to zero, implying that after this time there was no H-bond between the meuCl14 and the hMMP-2 catalytic domain. The vdW and electrostatic interaction energies during the dissociation of meuCl14 through the hMMP-2 catalytic domain are presented in Figure 7A. Again, fluctuations are observed for both of the vdW and electrostatic interaction energy curves during the SMD simulations. The highest peaks of vdW and electrostatics interaction energy occurred just after 8 ns. These are in harmony with the emergence time of the final peaks on the pulling force plot displaying the dissociation meuCl14 through the hMMP-2 catalytic domain (see Figure 4C). Apparently, after 8 ns the pulling force was used for the destruction of vdW and electrostatics interactions between the meuCl14 and the hMMP-2 catalytic domain, and the meucl14 would dissociate in a short time. Increasing the distance between the meuCl14 and the hMMP-2 catalytic domain with the passage time (Figure 7C) confirmed the preceding analysis. As shown in this figure, in the beginning, the distance curve was Horizontal, but shortly after 3 ns many of H-bonds were destroyed (see Figure 7B) and the first peak appeared on the pulling force plot (see Figure 4C). Simultaneously, the distance between the meuCl14 and the hMMP-2 catalytic domain increased, indicating that the dissociation of the main part of the ligand from the receptor has taken place. Furthermore, Figure 3C illustrates that the meuCl14 mostly dissociated from the hMMP-2 over 10 ns, yet a small part of it was still in interaction with hMMP-2; there would have been a total separation, if the simulation had continued.

Figure 8A depicts the changes of the vdW interaction and electrostatic energy during the dissociation of meuCl15 from the hMMP-2 catalytic domain. The curve in Figure 8B reveals that the numbers of H-bonds dwindled between 1 ns and 4 ns. As a result, after around 4ns, there was almost no H-bond left between the meuCl15 and the hMMP-2 catalytic domain. These time points are in agreement with the moments of the main peaks emergence in the pulling force plot of the meuCl15/hMMP-2 catalytic domain (Figure 4D). VdW and electrostatics interactions between the meuCl15 and the hMMP-2 catalytic domain also diminished with the decreasing number of H-bonds and, at last, return to zero at around 4 ns (Figure 8B), denoting that the meuCl15 completely dissociate from the hMMP-2. It can be also inferred from Figure 8C that the distance between the meuCl15 and the hMMP-2 catalytic domain became larger after around 4 ns. The total dissociation of meuCl15 at the end of simulation (10 ns) is further clear in Figure 3B.

Figure 9A presents the changes of the vdW interaction and electrostatic energy during the dissociation of meuCl16 from the hMMP-2 catalytic domain. No significant changes in the vdW and electrostatics interaction energy curves happened during the whole simulation time (Figure 9A). Noticeably, some fluctuations appeared in the number of H-bonds until around 4 ns (Figure 9B). After this time point, the number of H-bonds suddenly fell and finally sank to zero. It can be deducted that the main factor is H-bond when it comes to meuCl16/hMMP-2 catalytic domain binding. The pulling force returned to zero, as well, after 4ns (see Figure 4D) and the distance between the meuCl16 and the hMMP-2 catalytic domain increased after this time point (see Figure 8C), indicating a complete detachment of meuCl16 from hMMP-2. Figure 3B, clearly showed a complete dissociation of meuCl16 in hh10 ns.

## Conclusion

To sum up, in this study the 3-D homology model structures of three ClTxs of Iranian *M. eupeus *were determined and refined by MDFF simulation. 

Docking was made by hMMP-2 and Arginine residue was proposed as a key amino acid in interaction of ClTxs with hMMP-2.

The SMD simulations clearly demonstrate that the ClTx/hMMP-2 complexes are more stable than SC-74020/hMMP-2 complex. The current simulations provide a comparison of the pull forces required to undock the ClTx ligands from hMMP-2. It clearly reveals the most difficult hMMP-2 unbinding of meuCl14 compared to meuCl15 or meuCl16 or even the detachment of Chlorotoxin from the catalytic domain of hMMP-2; this is mainly due to the different electrostatic, vdW and H-bond interactions that the ligands form with hMMP-2. The pull force profiles of the undocking processes for the simulated complexes indicate that meuCl14/hMMP-2 complex is stabilized by vdW, electrostatic, and H-bond interactions, while the stabilization of meuCl15/hMMP-2 complex is mainly due to electrostatic and H-bond interactions; and H-bond interactions is the just stabilizing interaction in meuCl16/hMMP-2 complex.

This investigation in addition to clarifying the dissociation pathway of meuCl14, meuCl15, and meuCl16 from hMMP-2 catalytic domain also suggests meuCl14 as the most favorable ligand for hMMP-2 thanks to its stronger connection to hMMP-2. The information obtained here can be utilized to direct more exploration for better understanding of ClTxs’ mechanisms and behavior, especially for taking advantage of meuCl14 as a promising candidate in cancer drug delivery systems.
